# Fabrication of Mechanically Strong Silica Aerogels with the Thermally Induced Phase Separation (TIPS) Method of Poly(methyl methacrylate)

**DOI:** 10.3390/ma16103778

**Published:** 2023-05-17

**Authors:** Hainan Ma, Baomin Wang, Jiarui Qi, Yiheng Pan, Chao Chen

**Affiliations:** 1 College of Harbour and Coastal Engineering, Jimei University, Xiamen 361021, China; 2 Xiamen Key Laboratory of Green and Smart Coastal Engineering, Xiamen 361021, China; 3 School of Civil Engineering, Dalian University of Technology, Dalian 116024, China

**Keywords:** silica aerogel, poly(methyl methacrylate), thermally induced phase separation, porosity, mechanical properties

## Abstract

Constructing and maintaining a three-dimensional network structure with high porosity is critical to the preparation of silica aerogel materials because this structure provides excellent properties. However, due to the pearl-necklace-like structure and narrow interparticle necks, aerogels have poor mechanical strength and a brittle nature. Developing and designing lightweight silica aerogels with distinct mechanical properties is significant to extend their practical applications. In this work, thermally induced phase separation (TIPS) of poly(methyl methacrylate) (PMMA) from a mixture of ethanol and water was used to strengthen the skeletal network of aerogels. Strong and lightweight PMMA-modified silica aerogels were synthesized via the TIPS method and supercritically dried with carbon dioxide. The cloud point temperature of PMMA solutions, physical characteristics, morphological properties, microstructure, thermal conductivities, and mechanical properties were investigated. The resultant composited aerogels not only exhibit a homogenous mesoporous structure but also achieve a significant improvement in mechanical properties. The addition of PMMA increased the flexural strength and compressive strength by as much as 120% and 1400%, respectively, with the greatest amount of PMMA (*M*_w_ = 35,000 g/mole), while the density just increased by 28%. Overall, this research suggests that the TIPS method has great efficiency in reinforcing silica aerogels with less sacrifice of low density and large porosity.

## 1. Introduction

Silica aerogels’ low density, low thermal conductivity, high porosity, and specific surface area make them attractive for thermal and acoustic insulators, catalytic supports, adsorbents, sensors, radiation detectors, and drug delivery [1,2,3,4]. However, the same aggregate particulate structure and low volume percent solids that provide these properties limit the mechanical properties to the lesser cross-section of the neck structure connecting the particles [5,6]. Developing and designing lightweight silica aerogels with distinct mechanical properties is significant to extend their practical applications. 

To overcome the brittleness of silica aerogels, a variety of efforts have been reported to enhance the mechanical properties. One of the typical strategies to strengthen silica aerogels is to reinforce the necks in the aggregate with additional material. Early attempts utilized Ostwald ripening at a high pH, which strengthens the gel by redistributing mass to the contacts between particles [7,8,9]. Unfortunately, the reinforcement agent is still silica and remains a material with low tensile strength [10]. The alkyl or radicals of organosilanes, e.g., hexamethyldisilocane, hexamethyldisilazane, methyltriethoxysilane, and trimethylchlorosilane, were used to replace some hydroxyl and provide the monoliths extra strength and flexibility [11,12]. Precondensation of the silane precursor and sol-gel polymerization of the resulting high-molecular-weight poly(diethoxysiloxane) can also improve the mechanical properties of the aerogels [13]. Cross-linking with organic compounds or radical polymerization of monomers are effective techniques to obtain robust silica aerogels. Surface modification of the alcogel with reactive groups allows silicates [10], polyureas [14], polyurethanes [15,16,17], epoxies [18], polystyrene [19], and polymethacrylates [20,21,22] to be grown from or attached to the particles reinforcing the aerogel upon drying. Chemical vapor deposition and polymerization of cyanoacrylates [23,24,25] or silane monomers [26] can also be used to strengthen aerogels after supercritical drying. In one recent case, covalent attachment of polydimethylsiloxanes during supercritical drying was used to strengthen silica aerogels [27]. The polymers wrap the silica particles and strengthen the weak junctions. Unlike native silica aerogels with ultralow density and high specific area, many composite silica aerogels ultimately sacrificed some of the properties, e.g., density, porosity, surface area, and optical quality. Another well-known strategy for preparing mechanically strong silica aerogels is to introduce secondary reinforcement phases, such as glass fibers, nanoparticles, graphene, or nanotubes into the silica matrix [6,28,29,30,31,32,33,34,35,36,37]. In most cases, carbon nanofibers generate only weak physical bonds within the aerogel matrix, which may lead to short-term network integrity and shrinkage, thereby increasing the density [38], while graphene interacts with the silica matrix through hydrogen bonding without decreasing the density of the aerogels [39]. In recent years, many research works have focused on the silica–biopolymer hybrid aerogels owing to their flexibility, renewability, nontoxicity, and mechanical stiffness [40]. Cellulose-reinforced silica aerogels [41,42], silica–pectin hybrid aerogels [43], silk fibroin-reinforced silica aerogels [44,45,46], and chitosan-reinforced silica aerogels [47,48,49] were developed by adding biopolymers to silica backbones, which exhibit highly insulating properties and improved mechanical properties. However, extensive work at the pilot scale is required to evaluate if the hybrid material benefits offset the complex production process [38].

Here, we report a thermally induced phase separation method for depositing polymers onto silica particles. This approach relies on the poorer solubility of polymers and their phase segregation from theta solvents when cooled below their theta temperatures [50,51]. The TIPS method has been used to prepare polystyrene gels in which tetraethoxysilane (TEOS) was polymerized to form composite gels and aerogels [52]. Alternatively, TIPS can be heterogeneously nucleated by particles dispersed in the cooling solution, resulting in particles that are completely covered with the polymer [53]. While successfully applied to encapsulating catalyst particles, this approach has not been applied to silica alcogels.

In this work, thermally induced phase separation of PMMA from a mixture of ethanol and water was used to strengthen the skeletal network of aerogels. Since PMMA in ethanol/water solution has an upper critical solution temperature behavior, it could precipitate on the surface of silica particles during the cooling process [54]. Strong and lightweight PMMA-modified silica aerogels were synthesized via the TIPS method and supercritically dried with carbon dioxide. The resultant composited aerogels not only exhibit a homogenous mesoporous structure but also achieve a significant improvement in mechanical properties. This suggests that the TIPS method has great efficiency in reinforcing the mechanical strength while maintaining the unique properties of silica aerogels.

## 2. Materials and Methods

### 2.1. Materials

Tetraethoxysilane (98%) and two kinds of poly(methyl methacrylate) (PMMA-35k, *M*_w_ = 35,000 g/mole, PMMA-120k, *M*_w_ = 120,000 g/mole) were purchased from Sigma-Aldrich (St. Louis, MO, USA). Anhydrous ethanol was obtained from Decon Labs, Inc. (King of Prussia, PA, USA). Hydrochloric acid (37%) was purchased from EMD Millipore Co (Billerica, MA, USA). Ammonium hydroxide (29%) was supplied from Kanto Co (Portland, OR, USA). All the reagents were of analytical grade and used without further purification. A mixture of ethanol and deionized water (80 vol-%) was used to dissolve PMMA.

### 2.2. Synthesis of PMMA-Modified Aerogels via TIPS

TEOS (20.8 g, 0.100 mol), ethanol (18.4 g, 0.400 mol), and 1.8 mL of hydrochloric acid (0.04 M, 0.10 mol H_2_O) were combined to give 47.4 mL of clear colorless solution in a 100 mL flask. After stirring at 60 °C for 1.5 h, the solution was stored at −25 °C in the freezer until needed. A mixture of 5 mL of the solution (0.0105 mol Si) and 1 mL of ammonium hydroxide (0.25 M, 0.056 mol H_2_O) was poured into a polypropylene vial, making a final silicate concentration of 1.75 M. A transparent rigid gel formed within 17 min. After aging for 24 h at room temperature, the sample was removed from the vial into excess ethanol and placed in an oven at 50 °C for 24 h. Next, the ethanol in the silica gels was replaced by immersing each gel in 6 mL of an ethanol/water (80/20 vol) solution of PMMA at 60 °C for 3 days to ensure that the PMMA solution permeated into silica alcogels. At least six silica gels were prepared for each formulation.

A typical fabrication protocol for a PMMA solution is as follows. PMMA powder (0.030 g, 0.30 mmol) was completely dissolved in ethanol/water (80 vol-% ethanol, 6 mL) at 60 °C with stirring until the cloudy mixture turned transparent. To prepare the remaining samples, the following amounts of PMMA were dissolved in ethanol/water (80 vol-% ethanol, 6 mL): for 10 mg/mL, 0.060 g (0.6 mmol); for 15 mg/mL, 0.090 g (0.90 mmol); for 20 mg/mL, 0.120 g (1.20 mmol); for 40 mg/mL, 0.240 g (2.40 mmol); for 60 mg/mL, 0.360 g (3.60 mmol); for 80 mg/mL, 0.480 g (4.79 mmol). The phase separation, upon cooling in PMMA solutions to room temperature 20°C, the monolithic transparent gels turned opaque white. After being kept in PMMA solutions at 10 °C for 3 days, the aerogel monoliths were obtained by supercritical CO_2_ drying. The nomenclature of the samples is as follows: PS-X-Y, where PS refers to PMMA–silica aerogel, X is the average molecular weight of the PMMA in kDaltons, and Y is the concentration of the PMMA in solution used in the TIPS.

### 2.3. Characterization

Turbidity measurements were performed in a handheld spectrometer for UV-Vis measurements (Jaz Spectrometer, Ocean Optics Inc., Dunedin, FL, USA). Field-emission scanning electron microscopic (FESEM) images were recorded on a Nova Nanosem 450 instrument. A thin gold film was sputtered on the samples before the images were collected. Nitrogen adsorption/desorption isotherms were measured with a NOVA 4200e surface area and pore size analyzer (Quantachrome Instruments, Boynton Beach, FL, USA). The specific surface area of the sample was calculated by the Brunauer–Emmett–Teller (BET) method at the linear part (0.05 < P/P_0_ < 0.25) of the adsorption branch. Before the measurements, all samples were degassed at 60 °C for at least 6 h under vacuum. Infrared spectra were obtained in KBr pellets with a Nicolet FT-IR Spectrometer model Avatar 360. Thermogravimetric analysis was conducted with a TA Instrument under nitrogen and run at a temperature ramp rate of 10 °C/min. Thermal conductivities were determined from cylindrical specimens of monolithic aerogels using a Hot Disk TPS 2500S at 20 °C room temperature.

Flexural property measurements were assessed by a three-point flexural compression test with an Instron 5540 series single-column testing system with a 100 N load cell set with a 0.04 in/min crosshead speed according to ASTM D790 and ASTM C1684. Samples were cylinders, approximately 74 mm long by 8.5 mm in diameter. The distance between loads was 40 mm. Reported strengths are the average values from the analyses of 6 samples. Compression tests were conducted at a room temperature of 20 °C using a universal mechanical testing instrument (WDW3010, Kexin Co, Changchun, China), equipped with a 5 kN load cell. Since there are no standard test methods for aerogels, ASTM D695-10 was employed in this work. Cylindrical specimens were polished with fine-grade (#1000) sandpaper on all surfaces and checked by an L-square to ensure that the surfaces were smooth and parallel. According to ASTM standard, the slenderness ratio of the final samples for the compression test was 2:1, and the test speed was 1 mm/min.

## 3. Results and Discussion

### 3.1. Sol-Gel Polymerizations

Sol-gel polymerization of TEOS in ethanol was used in this case to permit TIPS modification with PMMA from ethanol–water solutions. The alcogels were prepared by the widely used two-step, acid- then base-catalyzed hydrolysis and condensation of TEOS [55,56]. Under these conditions, transparent, blue-tinted, silica alcogels formed within 17 min of adding aqueous ammonia. A concentration of TEOS was selected that would result in aerogels with a predicted density of 110 mg/mL. The unmodified silica gels were 139 mg/mL in density, consistent with a shrinkage of 20% during supercritical drying.

### 3.2. Turbidity Measurements of PMMA Solutions

**Figure 1:**
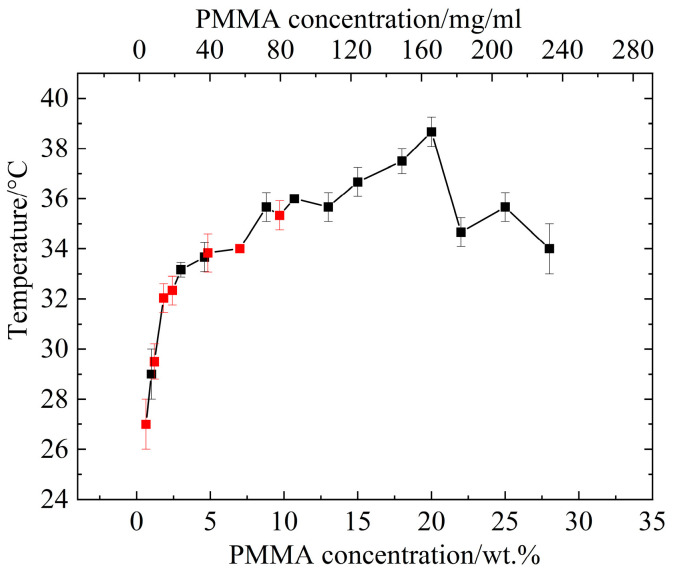
Cloud point temperatures for PMMA (0.6–28 wt%) solutions in ethanol/water; the red dots represent PMMA concentrations used in this paper.

To coat the colloidal particles making up the alcogel, PMMA had to be soluble in ethanol–water mixtures in the temperature range where silica alcogels are generally aged (50–60 °C) but insoluble at temperatures near 25 °C so that the ethanol–water could be eliminated without dissolving any PMMA deposited onto the gel. PMMA has upper critical solution temperature behavior in ethanol solutions [57]. At room temperature, PMMA will not dissolve in any water and ethanol mixtures. PMMA powder has maximum solubility in ethanol/water mixture (80/20 vol) at temperatures above 60 °C [57]. Since PMMA is insoluble in liquid and supercritical carbon dioxide [58,59], temperatures during the supercritical portion of the drying process are not important. Cloud points of PMMA in ethanol/water mixtures were confirmed using turbidimetry measurements on PMMA-35k solutions in ethanol/water solvent mixture (80/20 vol) prepared at 60 °C and cooled to a room temperature of 20 °C. When the phase separation occurred, the PMMA solutions became turbid, reducing light transmittance through the samples. The influence of PMMA-35k concentration on the cloud point temperature was evaluated from 0.6 wt.% to 28 wt.% in ethanol/water mixtures (80/20 vol). Figure 1 shows that the cloud points are below 50 °C and, for the most part, above 25 °C across the entire concentration range. Above the concentration of 9 wt.%, the PMMA solutions were too viscous to easily exchange into alcogels. Based on the graph of cloud point temperatures below, PMMA solutions between 0.6 wt-% and 8.8 wt-% provided a range of concentrations that would allow solvent exchange into the gel at 60 °C, coating out onto the gels by lowering to temperatures below room temperature and replacement of the ethanol–water solution with liquid carbon dioxide at 10 °C without fear of re-dissolving the PMMA.

### 3.3. Formation of PMMA-Modified Aerogels by TIPS

**Figure 2:**
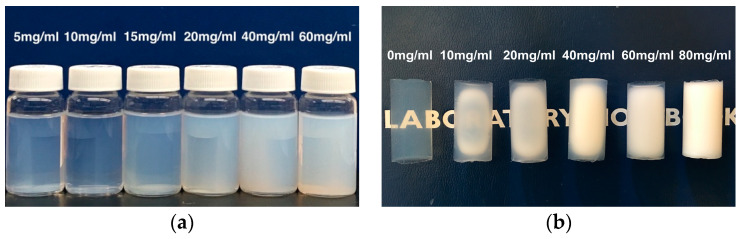
(**a**) Silica gels in suspensions of precipitated PMMA after TIPS and (**b**) aerogels with no PMMA (far left) and increasing amounts of PMMA going to the right.

After the silica alcogels had aged 24 h at room temperature and 24 h at 50 °C, they were immersed into solutions of varying amounts of PMMA in ethanol/water (80/20 vol) at 60 °C for 3 days to give the PMMA solution time to penetrate to the center of the transparent, blue-tinted gels. Upon cooling to a room temperature of 20 °C, the silica gels turned opalescent with the precipitation of the PMMA. The opalescence increased and the transparency decreased with the increasing concentration of PMMA in the solutions. The silica monolithic cylinders together with PMMA solutions were kept at 10 °C for 3 days before replacing the ethanol/water and any free precipitate particles with liquid carbon dioxide. Supercritical drying of the alcogels afforded aerogels that were increasingly white and opaque with greater concentration PMMA used in the TIPS. The cylindrical aerogels prepared for Figure 2 show some transparency along the surface of the aerogels with a lower PMMA concentration, suggesting that the lower cloud point temperature may have resulted in some re-dissolution of the polymer during processing. However, the effect diminished in aerogels prepared with higher PMMA concentrations.

Besides the increase in opacity, modification increased the density of the aerogels in proportion to the amount of PMMA used in the solution (Table 1). The densest modified aerogels were made with the 80 mg/mL solution of PMMA with a molecular weight of 35,000 g/mol. The PS-35k-80 aerogels were 0.178 g/cm. That is 39 milligrams of PMMA per cm^3^ or 22–23 milligrams per gram of aerogel. This constitutes a maximum increase in density of 28%. For most PMMA-modified aerogels, the increase in density was less than 15%. In comparison, chemical modification of alcogels with organic polymers results in aerogels with density increases of 100–800% [14,15,18,19,20,21]. With a higher molecular weight, the densities also increased with the concentration of PMMA-120k but only to a maximum density of 0.162 g/cm^3^.

The porosities of silica aerogels were calculated from the bulk density of PS-0-0 and the density of pure silica ($\rho_{s}=$2.19 g/cm^3^) according to Equation (1).

(1) $$P=\left(1-\frac{\rho_{0}}{\rho_{s}}\right)\times 100\%$$

The porosities of PMMA-modified aerogels were calculated with Equation (2).

(2) $$P=\left(1-\frac{\rho_{bulk}W_{p}}{\rho_{p}}-\frac{\rho_{bulk}(1-W_{p})}{\rho_{s}}\right)\times 100\%$$

In Equation (2), $\rho_{bulk}$ is the bulk density of modified aerogel, $\rho_{p}$ is the density of PMMA (for PMMA-35, the density is 1.17 g/cm^3^; for PMMA-120, the density is 1.188 g/cm^3^; *w_p_* is the mass of polymer incorporated into the silica aerogels, which was determined by the mass loss between 300 °C and 500 °C [20]; and $\rho_{s}$ is the density of pure silica.

PMMA content presented in Table 1 was calculated from the increase in densities and the mass losses in TGA. There was good agreement between the two methods for determining the mass of PMMA in the aerogels. Based on these masses, it is possible to calculate the percentage of PMMA in the polymer solution that precipitated onto the silica alcogel (the fourth column in Table 1). The amount varied from a low of 20% of the PMMA in solution ending up in the aerogel with PS-35k-5 to a high of 86.6% of the PMMA with PS-35k-15. The percentage of PMMA left in the aerogels peaked for both the 35K and 120K PMMA with lower concentrations. Despite the increase in aerogel density with PMMA concentration, PMMA-modified aerogels shrank less than the unmodified silica aerogels during drying. Linear shrinkage of silica aerogels during drying was 8–9%. The aerogels with the greatest amount of PMMA shrank only 6%.

**Figure 3:**
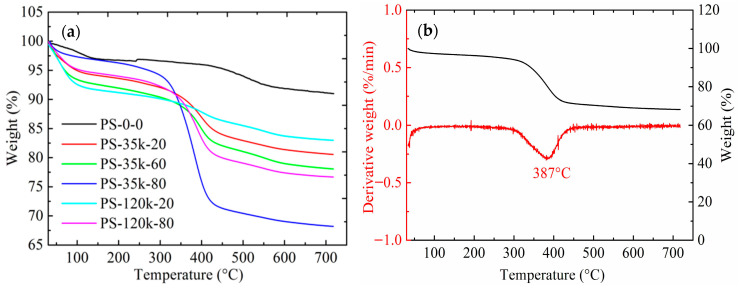
(**a**) TGA curves of PMMA-modified aerogels and (**b**) DTGA curve of sample PS-35k-80.

The amount of PMMA in the modified aerogels was determined by the difference in density compared to pure-silica aerogels and by the mass lost with heating as determined with thermal gravimetric analysis. The TGA curves of PMMA-modified aerogels are displayed in Figure 3. Unmodified aerogels (PS-0-0) lose a few percent mass below 100 °C from adsorbed moisture than 6.1% of mass above 400 °C with loss of water and alcohol from condensation reactions. In PMMA-modified samples, three thermal decomposition domains could be found in Figure 3a, and the weight losses in the three stages are listed in Table 2. In the first stage, consisting of the volatilization of the residual solvent and the absorbed water at 50–200 °C, the mass loss of PMMA-modified aerogels was below 7.5%. In the second stage, between 200 °C and 500 °C, the depolymerization of PMMA resulted in a large mass loss (23.7% of PS-35k-80). The amount of PMMA was determined from the mass loss between 300 °C and 500 °C because of the slow depolymerization of PMMA between 200 °C and 300 °C [21]. The weight loss in the third stage over 500 °C was mainly assigned to the condensation of -OH and the decomposition of a small amount of -CH_3_.

### 3.4. Aerogel Morphology

**Figure 4:**
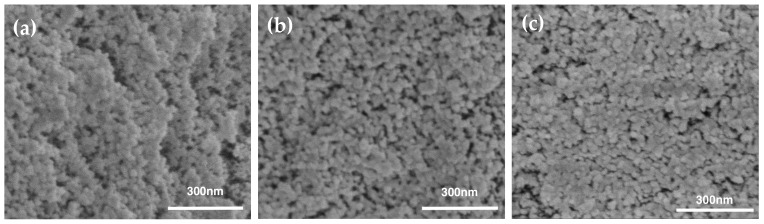

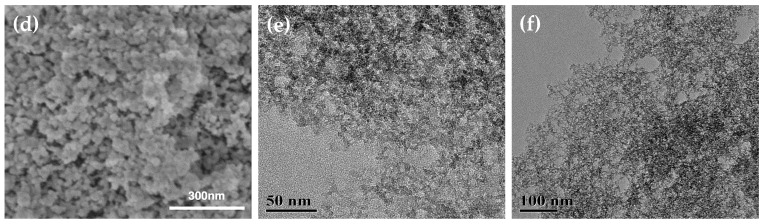
Microstructure of unmodified aerogel and PMMA-modified aerogels. (**a**) is pure-silica aerogel (ρ = 0.136 g/cm^3^), (**b**) is modified by PMMA-35k 20 mg/mL (ρ = 0.154 g/cm^3^), (**c**) is modified by PMMA-35k 80 mg/mL (ρ = 0.181 g/cm^3^), (**d**) is modified by PMMA-120k 60 mg/mL (ρ = 0.162 g/cm^3^), (**e**) is modified by PMMA-35k 10 mg/mL (ρ = 0.146 g/cm^3^), and (**f**) is modified by PMMA-120k 10 mg/mL (ρ = 0.143 g/cm^3^).

Aerogels are amorphous percolating networks of particles whose pores are tens of nanometers in diameter. Since they are over 90% porous (Table 1), aerogels typically exhibit the structures observed for the silica and PMMA-modified aerogels in Figure 4. From these representative micrographs, it is clear the PMMA polymer has minor effects on the three-dimensional interconnected network structure. It is consistent with the calculated porosities in Table 1, suggesting that the TIPS may have lowered the percentage porosity by 3% at most. At the highest PMMA concentration (Figure 4c), modified aerogels display a coarser network than pure-silica aerogel. For all investigated series of modified aerogels, increasing the polymer concentration leads to fine microstructures with larger particles, in agreement with the bulk density analysis in Table 1.

**Figure 5:**
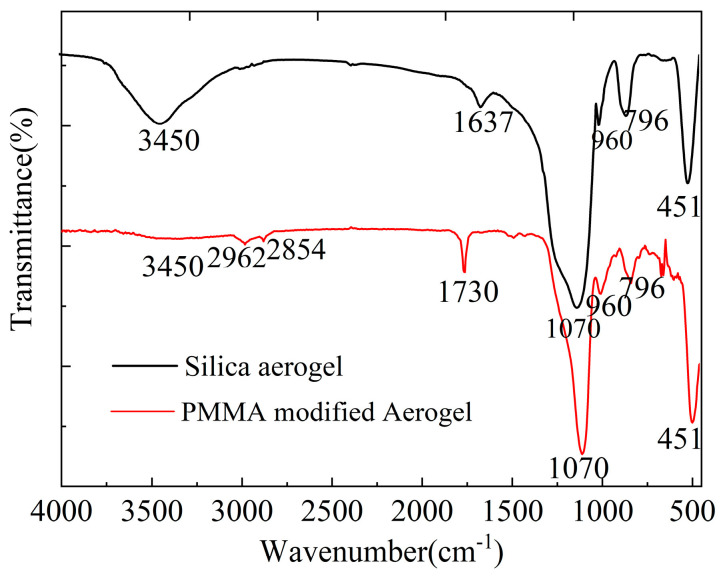
FT-IR spectra of unmodified aerogel and PMMA-modified silica aerogel.

The presence of PMMA in the TIPS-modified aerogels was confirmed by an infrared spectroscopic comparison of pure-silica aerogels and PMMA-modified aerogels in Figure 5. PMMA-modified samples exhibit the characteristic ester C=O stretch at 1730 cm^−1^, making it easy to distinguish from unmodified silica aerogels [21]. The peaks at 1070 cm^−1^, 796 cm^−1^, and 451 cm^−1^ are due to the asymmetrical stretching vibration, symmetric stretching vibration, and bending vibration of Si-O-Si groups, respectively [60]. The broad absorption peaks at 3450 cm^−1^ and 960 cm^−1^ due to -OH groups and the hydrogen-bonded water at 1637 cm^−1^ on the surface of silica aerogels [61] are significantly reduced in the PMMA-modified aerogels, indicating that the new materials are relatively hydrophobic compared to silica aerogels.

### 3.5. Pore Structure Analysis

**Figure 6:**
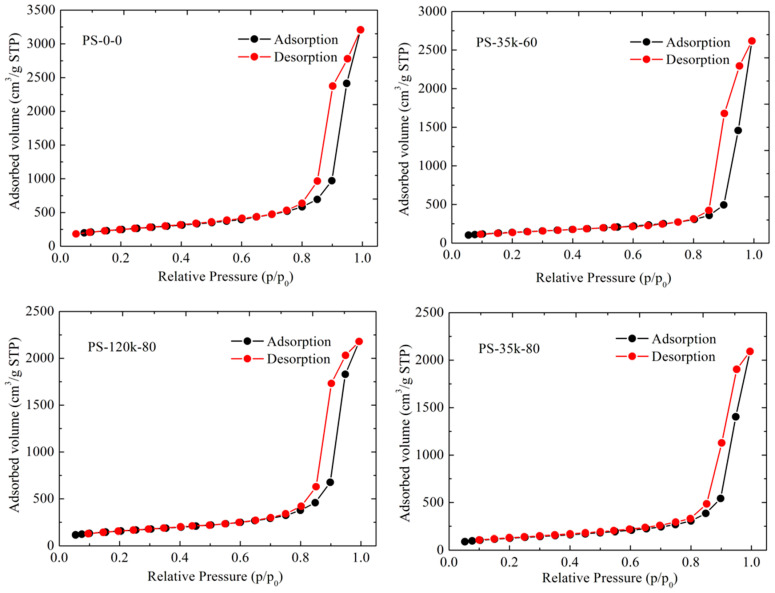
N_2_ adsorption–desorption isotherms of pure aerogels and PMMA modified aerogels.

**Figure 7:**
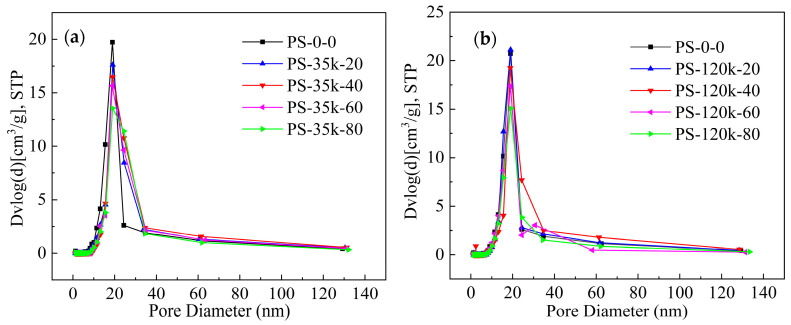
Pore size distribution of (**a**) PMMA-35K-modified aerogels and (**b**) PMMA-120K-modified aerogels.

Nitrogen sorption analysis was used to characterize the influence of PMMA on the specific surface area and pore size distribution of the aerogels. It is clearly observed from Figure 6 and Figure 7 that the resulting aerogels display a typical IV isotherm with a distinct capillary condensation step at the relative pressure of 0.7–1.0 and the narrow pore size distributions, demonstrating the mesoporous three-dimensional network structure that is composed of aggregated nanoparticles [62,63]. The randomly interconnected mesoporous structural feature could be further confirmed by the SEM and TEM images in Figure 4. Specific surface areas, pore volumes, and average pore sizes of the silica aerogels and the PMMA-modified aerogels are given in Table 3.

Modifying silica aerogel with PMMA solution via the TIPS method resulted in a decrease in the Brunauer–Emmett–Teller (BET) surface area [64] from 874 m^2^/g for a 0.139 g/cm^3^ pure aerogel to 455 m^2^/g for the 0.178 g/cm^3^ PMMA-35k-modified aerogel with a polymer content of 80 mg/mL. A decrease in surface area is commonly observed with additive approaches to strengthening silica aerogels growing organic polymers off the colloidal network. Although the surface area decreases by 48% with PS-35k-80, it is still less than that reported for silica aerogels reinforced by surface modification or cross-linking with organic polymers where surface areas decrease by 75% or more [14,15,18,19,20,21]. Pore diameters (*D*_BJH_) were calculated using the Barrett–Joyner–Halenda (BJH) model [65] applied to the desorption branch of the isotherms in Figure 6. In general, the aerogel mesopores were 17–18 nm in diameter, consistent with the size of the pores observed in the SEM images in Figure 4. There was no indication of pore coarsening observed with aerogels that had been aged longer before drying. The calculated pore volumes decrease with increasing PMMA content. One explanation of decreasing surface area and pore volume but constant pore size is that the PMMA is deposited in micropores that are not included in BJH calculations. This was supported by the decrease in micropore volumes, calculated using Nonlocal Density Functional Theory (NLDFT) method, with increasing PMMA.

### 3.6. Thermal Conductivity

**Figure 8:**
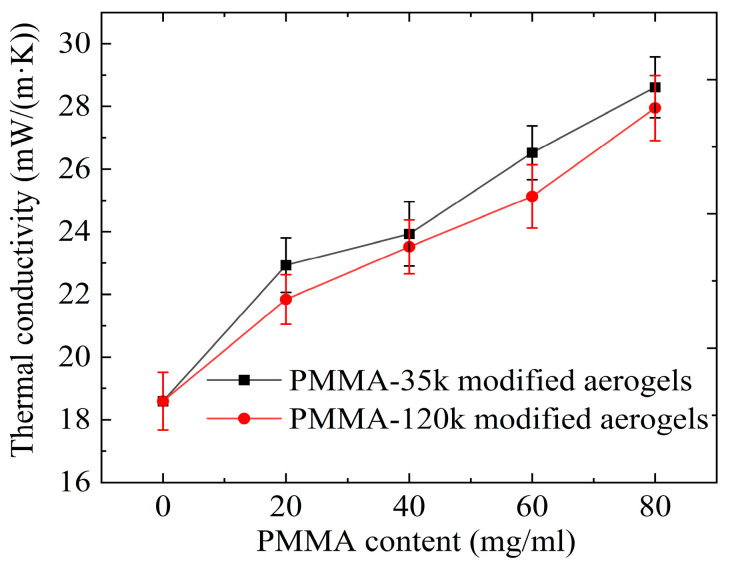
Thermal conductivity of PMMA-modified silica aerogel.

The thermal conductivity was measured on a monolithic 40 × 40 × 12 mm^3^ cylinder by the transient plane source method at 23 °C. Figure 8 shows the thermal conductivity of modified aerogels with different PMMA concentrations. Increasing the PMMA concentration increases the thermal conductivity owing to the higher solid conductivity. The introduction of PMMA partially fills the microporous structure and increases the average pore diameter, thereby increasing the gas phase thermal conductivity of the modified aerogel. However, the increase is minor, and the whole series of PMMA-modified aerogels show good thermal insulation performance. The sample with a PMMA-35k concentration of 80 mg/mL showed the highest thermal conductivity, 28.61 mW/(m·K).

### 3.7. Mechanical Properties of Aerogels

**Figure 9:**
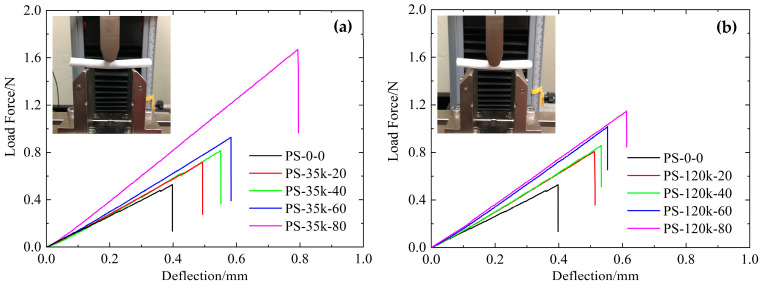
Load–deflection characteristic of (**a**) PMMA-35k-modified aerogels and (**b**) PMMA-120k-modified aerogels.

Flexural and compressive testing of the aerogels was conducted to determine how TIPS modification of aerogels changed their mechanical properties. Flexural testing of silica and PMMA-modified aerogels were performed on monolithic cylindrical samples using a three-point bending method according to ASTM D790 and ASTM C1684. The typical load–deflection curves are shown in Figure 9, and the calculated flexural strengths and moduli are shown in Table 4. The flexural strength and modulus of the pure-silica aerogels are similar to those previously reported for aerogels near this density [21,22,24]. With increasing polymer content, both PMMA-35k- and PMMA-120k-modified aerogels were strengthened to withstand greater loads.

**Figure 10:**
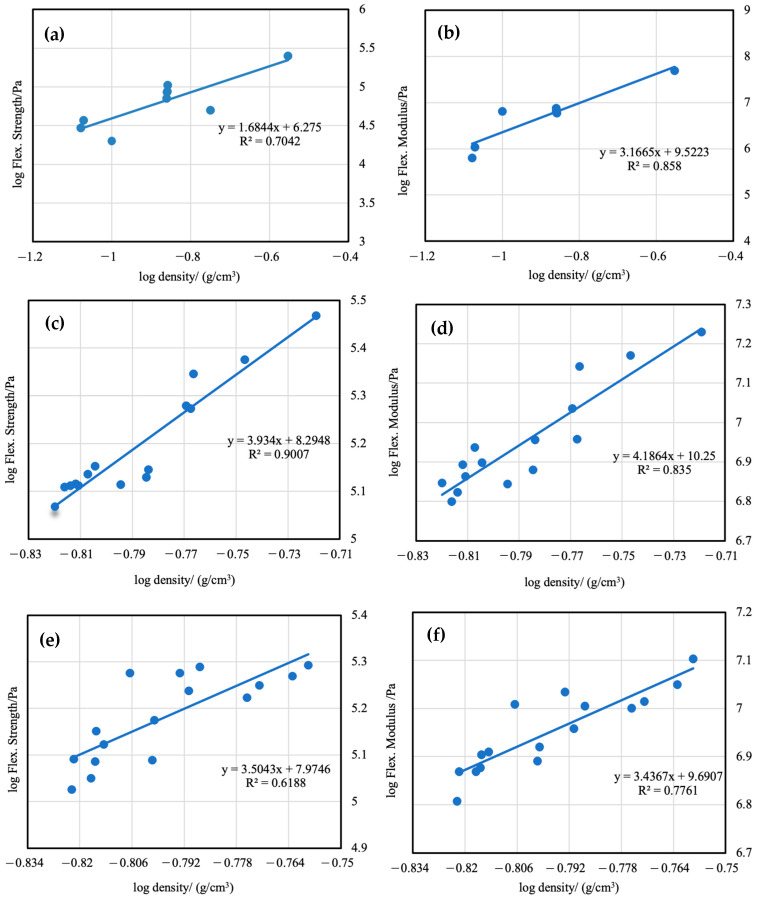
Flexural strength and flexural modulus versus bulk density of unmodified silica aerogels (**a**,**b**), PMMA-35k-modified aerogels (**c**,**d**), PMMA-120k-modified aerogels (**e**,**f**).

The relationship between the strength and modulus of silica aerogels and bulk density can be described by the power law. The log–log plots of flexural strength and modulus versus bulk density were analyzed in Figure 10. The results indicate that the flexural strength and modulus increase faster by the addition of PMMA into the preformed skeletal framework than by using more TEOS in the sol. Although the mechanical properties of both modified and unmodified aerogels would increase with the rise in density, PMMA-modified aerogels are stronger than silica alone at equivalent densities. This is because the increase in the mass of silica in aerogels means that there will be a greater number of interparticle necks in the skeleton, and the narrow junctions are the main reason for the poor mechanical properties of aerogels. The PMMA polymer introduced into the gels by the TIPS method was deposited and wrapped on the surface of silica particles, which increases the area of the neck region between the particles, thereby improving the mechanical properties of the aerogels.

For PMMA-35k-modified aerogels, the flexible strength and modulus significantly increased by 120% and 125%, respectively, with 80 mg/mL PMMA, while the density increased by 28%. These samples were able to survive twice the deflection required to break the pure-silica aerogels. This strengthening is attributed to the impregnation of polymer into micropores of secondary particles, which increases the solid contacts between secondary particles. Modification of silica aerogels with 80 mg/mL of PMMA-120k increases the flexible strength and flexible modulus by 72% and 97%, respectively. The smaller increase may be due to the size exclusion of polymers by micropores. Overall, mechanical reinforcement correlates well with the bulk density and micropore volume calculated by the DR method.

**Figure 11:**
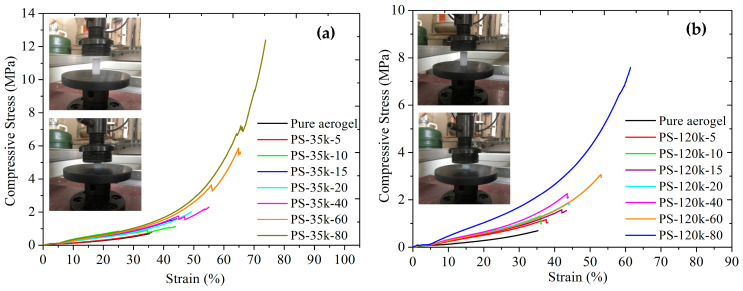
Compressive stress–strain curves of (**a**) PMMA-35k-modified aerogels and (**b**) PMMA-120k-modified aerogels.

Compressive stress–strain curves of representative PMMA-modified aerogels with various polymer contents are shown in Figure 11. Silica aerogels exhibited a relatively linear compression curve with failure at 0.74 MPa and a modulus of 0.78 MPa, comparable to earlier reports [27,65]. The ultimate strength and strain of modified aerogels increase with increasing PMMA content in a fashion similar to increasing the organic component in formaldehyde-resorcinol-silica composite aerogels [66]. The strain at failure of PMMA-modified aerogel is double that of the silica aerogel without PMMA. In contrast, the strengthening of silica aerogels through surface modification with polydimethylsiloxane during supercritical CO_2_ drying increased the ultimate compressive strength but decreased the ultimate strain [27].

**Figure 12:**
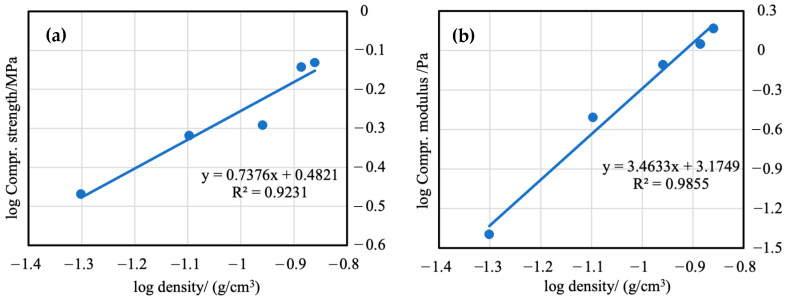

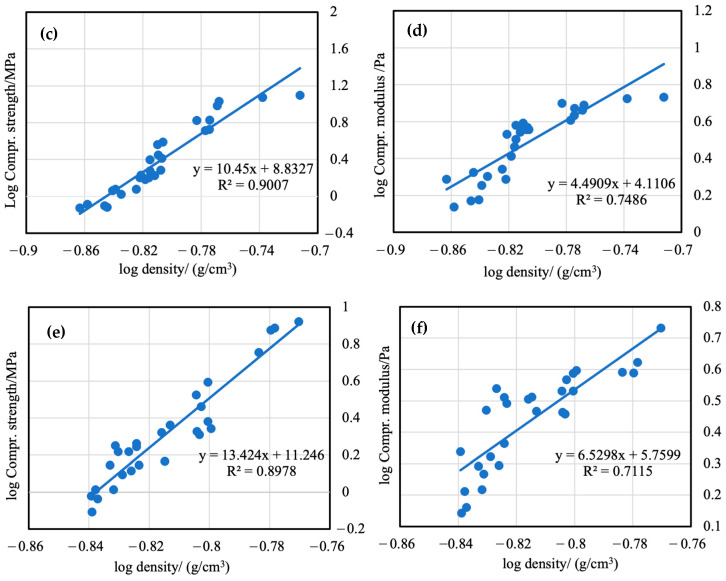
Compressive strength and compressive modulus versus bulk density of unmodified silica aerogels (**a**,**b**), PMMA-35k-modified aerogels (**c**,**d**), PMMA-120k-modified aerogels (**e**,**f**).

Most impressively, the compressive strength increased by 1400%, and the compressive modulus increased by 547% over the untreated silica aerogels with 80 mg/mL PMMA-35k treatment. As with the flexural measurements, it is evident that the PMMA-35k provides superior strengthening to the PMM-120k. The PS-35k-80 (0.178 g/cm^3^) sample failed at 73% strain under ~12 MPa compression stress, while the pure aerogel (0.136 g/cm^3^) just failed at 35% strain under ~0.7 MPa compressive stress. For the same polymer concentration, PS-120k-80 could only sustain compression to 62% strain and ~7.5 MPa stress. In Figure 12, ultimate compression stress versus bulk density is plotted. The final compression stress correlates well with the bulk density, and the slopes of PMMA-modified aerogels are much higher than that of pure aerogels. It means that a very slight increase in bulk density caused by the increase in the PMMA concentration resulted in a large increase in the compressive strength and modulus.

## 4. Conclusions

In conclusion, mechanically strong and lightweight PMMA-modified silica aerogels have been successfully synthesized via the thermally induced phase separation method and supercritically dried with carbon dioxide. The resulting aerogels show a homogenous mesoporous structure and a coarser skeleton network, leading to a combination of low density (0.139–0.180 g/cm^3^), uniform pore size (mainly <30 nm), high porosity (90.1–93.4%), low thermal conductivity (19.20–28.61 mW/(m·K)), and outstanding mechanical properties. At a concentration of 80 mg/mL PMMA, the PMMA-35k-modified aerogels demonstrated a significant increase in flexural strength (120%) and modulus (125%) with only a 28% increase in density. Moreover, the PMMA-35k-modified aerogels exhibited an even greater improvement in compressive strength (1400%) and modulus (547%). Electron microscopy observation and pore size analyses revealed that the TIPS method for modifying silica aerogels with PMMA is favorable for maintaining the three-dimensional reticulated mesoporous structure. This study demonstrates the potential of thermally induced phase separation to reinforce the mechanical properties of silica aerogels with poly(methyl methacrylate), which offers a new way to develop robust polymer-modified silica aerogels with less environmental burden. Further investigations on the synthesis of polymer-modified aerogels through the TIPS method and their applications are being conducted in our laboratory.

## Figures and Tables

**Table 1:** Summary of the physical characteristics of pure aerogels and PMMA-modified aerogels.

Samples *	Bulk Density (g/cm^3^)	Porosity (%)	Percent PMMA Incorp (%) **	PMMA Content Densities (g/g) ***	Mass Loss TGA (%)	PMMA Content TGA (g/g)
PS-0-0	0.139 ± 0.002	93.7	0	0	9.1	0
PS-35k-5	0.140 ± 0.002	93.4	20.0	0.0071	12.2	0.0273
PS-35k-10	0.147 ± 0.004	93.0	80.0	0.0576	14.6	0.0459
PS-35k-15	0.152 ± 0.003	92.8	86.6	0.0855	15.3	0.0587
PS-35k-20	0.153 ± 0.004	92.5	70.0	0.0915	19.5	0.0912
PS-35k-40	0.154 ± 0.001	92.4	37.5	0.1080	21.0	0.1070
PS-35k-60	0.165 ± 0.004	91.7	43.3	0.1580	22.9	0.1142
PS-35k-80	0.178 ± 0.010	90.1	48.7	0.2190	31.8	0.2370
PS-120k-5	0.143 ± 0.002	93.2	80.0	0.0280	12.5	0.0282
PS-120k-10	0.145 ± 0.003	93.1	60.0	0.0414	13.1	0.0329
PS-120k-15	0.151 ± 0.005	92.8	80.0	0.0795	13.2	0.0354
PS-120k-20	0.153 ± 0.005	92.7	70.0	0.0920	17.0	0.0461
PS-120k-40	0.156 ± 0.003	92.4	42.5	0.1090	18.1	0.0552
PS-120k-60	0.159 ± 0.003	92.0	33.3	0.1260	19.8	0.1029
PS-120k-80	0.162 ± 0.002	91.7	28.7	0.1420	23.4	0.1491

* The samples are labeled as PS-X-Y, where PS refers to PMMA–silica aerogel, X is the number average molecular weight of the PMMA, and Y is the concentration of the of the PMMA in the solution used in the TIPS. ** The percent PMMA incorporated is based on the mass of PMMA on the aerogel divided by mass of PMMA in the original solution. *** PMMA content densities are the grams of PMMA per gram of aerogel determined from densities.

**Table 2:** Summary of weight losses for all three domains.

Samples	Mass Loss TGA (%)	Mass Loss between 50–200 °C	Mass Loss between 200–500 °C	Mass Loss over 500 °C
PS-35k-20	19.5	5.0	9.1	5.2
PS-35k-40	21.0	5.2	7.8	7.9
PS-35k-60	22.9	5.3	9.3	7.5
PS-35k-80	31.8	6.1	23.7	4.3
PS-120k-20	17.0	3.8	4.6	4.6
PS-120k-40	18.1	7.8	5.5	7.3
PS-120k-60	19.8	5.3	10.2	4.8
PS-120k-80	23.4	4.8	13.2	4.4

**Table 3:** Pore size analysis of pure aerogels and PMMA-modified aerogels.

Sample	*S_BET_* (m^2^/g)	*S_NLDFT_* (m^2^/g)	*V_pore,BJH_* (cm^3^/g)	*V_pore,NLDFT_* (cm^3^/g)	*D_pore,BJH_* (nm)
PS-0-0	880	933	5.076	4.758	17.67
PS-35k-15	731	775	4.944	4.286	17.68
PS-35k-20	639	700	4.210	3.769	17.72
PS-35k-40	519	579	3.668	3.767	17.66
PS-35k-60	495	583	3.326	3.607	17.69
PS-35k-80	455	541	3.320	2.929	17.71
PS-120k-15	706	825	4.497	4.306	17.68
PS-120k-20	695	804	4.635	4.225	17.65
PS-120k-40	663	773	5.069	4.100	17.67
PS-120k-60	607	676	4.185	3.816	17.71
PS-120k-80	548	652	3.535	3.202	17.70

*S_BET_*: multipoint BET surface area; *V_pore,BJH_*: BJH method cumulative volume; *V_pore,NLDFT_*: NLDFT method cumulative pore volume; *D_pore,BJH_:* BJH method pore diameter (desorption branch).

**Table 4:** Mechanical properties of pure aerogels and PMMA-modified aerogels.

Sample	Density (g/cm^3^)	Flex. Strength (10^5^ Pa)	Flex. Modulus (MPa)	Compr. Strength (MPa)	Compr. Modulus (MPa)
PS-0-0	0.139 ± 0.002	0.98 ± 0.20	5.61 ± 0.24	0.74 ± 0.17	0.78 ± 0.31
PS-35k-20	0.153 ± 0.004	1.13 ± 0.13	7.10 ± 0.71	1.97 ± 0.41	3.53 ± 0.59
PS-35k-40	0.154 ± 0.001	1.25 ± 0.11	6.98 ± 0.96	3.21 ± 0.67	3.75 ± 0.83
PS-35k-60	0.165 ± 0.004	1.39 ± 0.24	7.58 ± 0.17	5.96 ± 0.82	4.52 ± 0.47
PS-35k-80	0.178 ± 0.010	2.15 ± 0.46	12.60 ± 0.25	11.15 ± 0.127	5.05 ± 0.82
PS-120k-20	0.153 ± 0.002	1.37 ± 0.30	8.01 ± 0.23	1.79 ± 0.35	3.22 ± 0.22
PS-120k-40	0.156 ± 0.005	1.48 ± 0.22	8.66 ± 0.11	2.91 ± 0.43	3.33 ± 0.19
PS-120k-60	0.159 ± 0.005	1.52 ± 0.35	8.63 ± 0.16	3.07 ± 0.76	3.54 ± 0.30
PS-120k-80	0.171 ± 0.003	1.69 ± 0.27	11.06 ± 0.12	7.31 ± 0.114	4.34 ± 0.72

Data are the average of at least six stress–strain analyses for every sample formulation.

## Data Availability

Raw data are available upon request.
